# Efficient state of charge estimation in electric vehicles batteries based on the extra tree regressor: A data-driven approach

**DOI:** 10.1016/j.heliyon.2024.e25949

**Published:** 2024-02-09

**Authors:** Sadiqa Jafari, Yung-Cheol Byun

**Affiliations:** a Department of Electronic Engineering, Institute of Information Science & Technology, Jeju National University, Jeju 63243, South Korea; b Department of Computer Engineering, Major of Electronic Engineering, Jeju National University, Institute of Information Science & Technology, Jeju 63243, South Korea

**Keywords:** Electric vehicles, State of charge prediction, Extra tree regressor, Light gradient boosting, Driving cycle, Battery data

## Abstract

Global warming, a significant outcome of climate change, exerts detrimental effects on the daily lives of individuals and industries. As a result, there is an increased demand for Electric Vehicles (EVs) to reduce carbon emissions contributing to climate change. This shift underscores the critical need for accurate estimation of the State of Charge (SoC) in battery systems, which is essential for optimizing EVs' performance and ensuring effective energy utilization. This paper introduces a methodically constructed and tested SoC prediction model utilizing a comprehensive dataset derived from various driving cycles and battery records. The battery performance of EVs was assessed in our study. The essence of our innovation resides in the meticulous choice of representative driving cycles, effectively replicating real-world conditions. This methodology improves the model's capacity to apply to various driving patterns and conditions. During these cycles, a comprehensive set of battery data, encompassing voltage, current, temperature, and SoC, was systematically documented to facilitate thorough analysis. To achieve superior accuracy and robustness, our predictive model considers the strengths of the Extra Tree Regressor (ETR) and Light Gradient Boosting algorithms. Our experimental results demonstrate the remarkable performance of the ETR model in predicting SoC, surpassing the LightGBM model. The ETR model exhibited higher $R^{2}$ values of 0.9983 and lower Root Mean Square Error (RMSE) of 0.62, Mean Absolute Error (MAE) of 0.085, and Mean Squared Error (MSE) of 0.39 values, underscoring its superiority. The research emphasizes the considerable significance of battery capacity in effectively predicting the SoC of EVs. Our research highlights the significant importance of battery capacity in accurately forecasting the SoC of EVs. The proposed model facilitates accurate SoC predictions, improving energy management in EVs to optimize battery utilization and support informed decisions toward sustainable mobility.

## 1 Introduction

Since their development decades ago, Electric Vehicles (EVs) have increased and are now a widespread mode of transportation. Compared to gas-powered vehicles, EVs provide unrivaled advantages such as quick acceleration, almost little noise, and minimal pollution [1]. With a breakthrough in creating renewable energy storage and conversion technologies, performances continue to improve [2]. There is always some uncertainty when using battery-powered equipment, but there is minimal opportunity for such uncertainty in a motorized vehicle. Humans have been testing and using gas-powered cars for over a century, but EVs are still in their infancy and are being developed. There are several objections to owning an EV, although the markets for EVs have grown. Range concern is the main barrier preventing consumers from adopting electric automobiles [3]. The most effective energy storage devices that can deliver power fast are Lithium-Ion Batteries (LIBs). LIBs are arranged into multiple modules to provide the vehicle electricity and coupled in series, parallel, or mixed fashions. In each EV, hundreds or thousands of LIBs are often fitted [4]. To extend the battery's life and performance and improve security, the Battery Management System (BMS) in the cars serves as an EV and thermal management system. The primary responsibilities of the BMS are to control the temperature of the batteries to prevent thermal runaway or explosion, monitor the voltage and current of the batteries to prevent overcharging or over-discharging, diagnose and detect faults, and estimate the batteries' remaining energy. Despite advancements in BMS technology, the rest of the mileage range remains unpredictable because of the deterioration and instability of the batteries in diverse situations. The State of Charge (SoC) is characterized by a ratio of residual capacity to the battery's available capacity [5]. It has been reported that several methods can be used to estimate the battery status in EVs. They can generally be divided into three types: 1) Measurements based on physical properties, such as coulomb counting, open circuit voltage, and electrochemical impedance spectroscopy; 2) statistical derivations, such as Kalman Filter (KF) and Particle Filter (PF); and 3) data-driven techniques, primarily Machine Learning (ML) and deep learning [6]. This study addresses the crucial need for precise SoC prediction in EV batteries. In order to achieve accurate and reliable predictions, a robust prediction model is developed and validated. The selection of the Extra Tree Regressor (ETR) and LightGBM algorithms is driven by their proven capabilities in handling complex and high-dimensional datasets, making them well-suited for battery SoC estimation. The ETR algorithm utilizes ensemble learning, combining multiple decision trees to minimize overfitting and enhance robustness. On the other hand, LightGBM, a gradient boosting framework, optimizes the boosting process efficiently, enabling faster and more scalable performance. These algorithmic strengths make ETR and LightGBM ideal candidates for real-time and resource-intensive tasks like battery SoC estimation. By leveraging the capabilities of ETR and LightGBM, our model aims to provide superior accuracy and generalization across various driving cycles and battery datasets. Combining these algorithms ensures a comprehensive data analysis, enabling the model to capture intricate relationships within the battery system. Our work creates several critical contributions to the area of battery performance estimation in EVs, which can be summarized as follows:

• We develop a novel technique based on ETR and LightGBM for the SoC battery estimation in EVs. The SoC estimation accuracy and reliability for EV batteries improve with this approach.
• Our research involves a detailed evaluation and comparison of our proposed model against various other models in the field. This comprehensive analysis enables us to pinpoint the most effective model for SoC estimation in EVs.
• We demonstrate our proposed method's superiority over the existing one on established battery heating datasets by using various models to train and validate it and by rigorously testing optimal models through experimentation.
• We show the superior performance of the ETR model in SoC prediction for the battery heating in the EVs.

The rest of the paper is organized as follows: section 2 provides a concise overview of the existing literature pertaining to the proposed design framework. Then, we explain the proposed method and machine learning models in sections 3, and in section 4, we present the battery data source. The implementation process, data processing, data description, result, and performance evaluation are in section 5; finally, we complete this paper in the conclusion and future work section.

## 2 Related work

**Figure 1:**
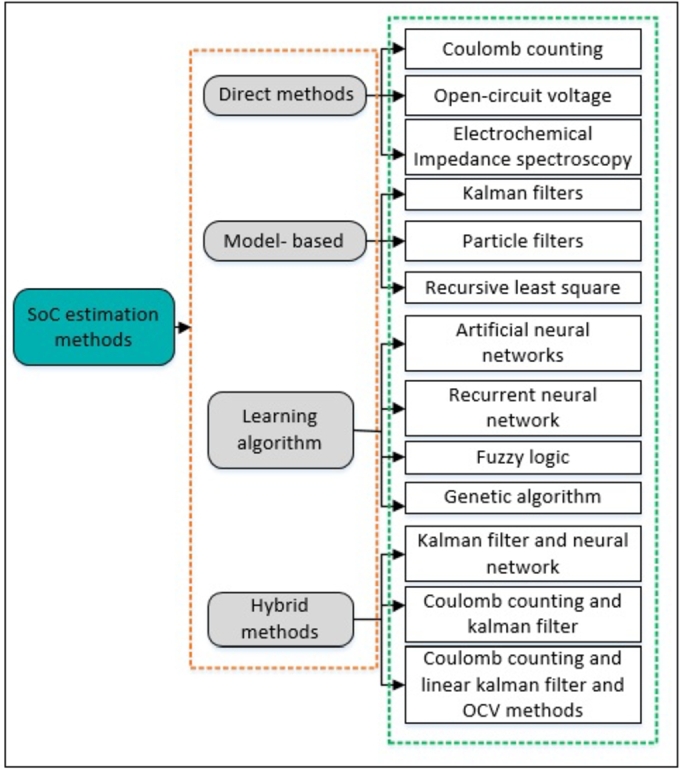
SoC estimation methods.

Amid the global evolution towards renewable and clean energy solutions, driven by the fossil fuel problem and environmental considerations, improving the energy industry with sustainable options has evolved critically [7], [8], [9]. EVs, renowned for their exceptional energy efficiency and complete absence of pollutants, have garnered considerable attention on a global scale [10]. LIBs, which function as key energy storage devices and are essential for dependable and efficient operation, are vital to their operation [11], [12]. The precise administration of LIBs, specifically in estimating the SoC, is imperative for achieving optimal performance [13]. In practical contexts, the SoC pertains to the proportion of the available capacity relative to the rated capacity, offering an intuitive indication of the remaining viable power within LIBs [14]. Recent scholarly research has delved into a myriad of methodologies for estimating the SoC of electric vehicle batteries, a critical aspect of energy management in EV technology [15], [16], [17]. As delineated in Fig. 1, these methodologies are categorized into four principal groups. The first group, direct methods, encompasses techniques such as coulomb counting and open-circuit voltage measurement, which are fundamental yet straightforward. Model-based methods, the second group, involve Kalman filters, particle filters, and recursive least squares for predictive SoC analysis. The third group, learning algorithms, corresponds to data-driven approaches and includes advanced computational models such as neural networks and fuzzy logic. The final group, hybrid methods, synergizes the aforementioned approaches, combining, for instance, the predictive precision of model-based methods with the adaptive learning capacity of neural networks to refine accuracy and reliability in SoC estimation [18], [19].

Hybrid neural network models, such as the Convolutional Neural Network-Bidirectional Weighted Gated Recurrent Unit (CNN-BWGRU) hybrid, have advanced recently for temperature-sensitive SoC estimation [20], [21]. However, a research gap becomes apparent when considering the management of LIBs in different settings, such as severe temperatures or varying driving profiles. Previous research studies have commenced efforts to tackle these obstacles by employing innovative techniques like Singular Filtering-Gaussian Process Regression-Long Short Term Memory (SF-GPR-LSTM), specifically emphasizing enhancing performance at low temperatures. However, there is still a want for methodologies that possess both adaptability across different operational settings and ease of implementation [22], [23]. This study addresses the above research gap by incorporating deep learning methodologies into analyzing real-world driving data. The methodology we propose differs from current methodologies as it provides a more versatile and pragmatic solution for a wide range of operational circumstances without requiring an extensive understanding of battery chemistry or dependence on supplementary filters [24], [25], [26], [27] In this paper, battery capacity estimation using data-driven techniques has shown promising results, but there are restrictions on their application scenarios and the time needed for feature determination. The research has proposed a data-centric approach for estimating capacity, which may be utilized in the high SoC range. It necessitates only 10 minutes of relaxation voltage data. Experiments employing commercial batteries have investigated the interplay between relaxation voltage, battery aging, and charging cut-off SoC [28]. By focusing on a technique that balances accuracy, generalizability, and simplicity, we aim to contribute to the area by providing a robust and versatile SoC estimation tool in various real-world systems. Although plenty of research was done based on the SoC estimation, Table 1 summarizes some of the latest studies on the SoC estimation of batteries with ML algorithms.

**Table 1:** Recent works on SoC estimation of batteries based on the ML techniques.

Trends	Approach	Objective	Benefit
SoC [29]	Multimode Ensemble Support Vector Regression (ME-SVR)	Enhance accuracy, improve stability, increase generalization.	Improved battery management, extended battery lifespan, optimized EV performance
SoC [30]	Machine learning algorithms	SoC estimation, optimize battery performance parameters	Improved durability and reliability of battery management systems, accurate SoC estimation, enhanced battery performance
Increasing demand for li-ion batteries, SoC [31]	Support Vector Regression and Gradient Boosting Techniques (SVRGT)	Accurate SoC estimation, comparison, performance, Efficiency	Improved battery management, reliable estimation
Data-driven SoC estimation for li-ion batteries [32]	Improved Whale Optimization (IWOA-Ada)Boost-Elman	Develop a SoC estimation, prediction accuracy	Accurate and stable SoC estimation
SoC estimation, li-ion batteries [33]	Automotive simulations and multi-physics	Simulation, SoC dynamic response	Precise SoC estimation, Improved battery performance

## 3 Methodology

This paper presents an innovative system architecture for the suggested method of using machine learning, which effectively combines user input with real-time and historical battery data from EVs. The presented methodology autonomously acquires data on battery charging and discharging while the vehicle is in operation, thereby obviating the requirement for active user involvement and augmenting the system's usability and efficiency.

**Figure 2:**
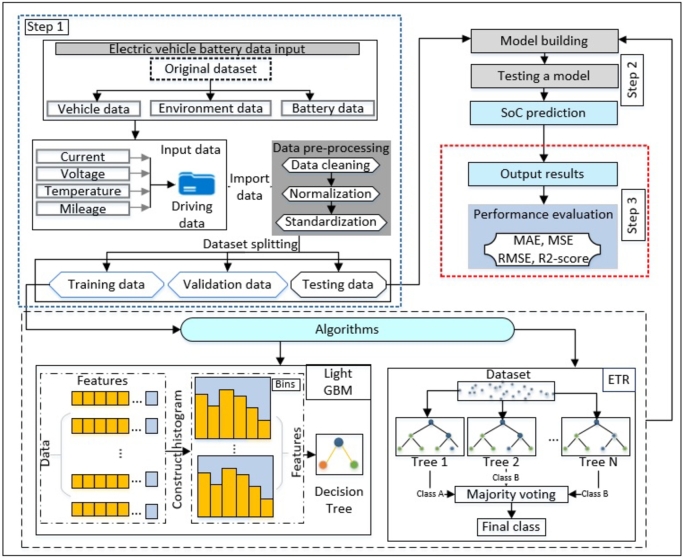
The general design of the suggested approach.

The essential aspect of our unique approach for estimating the SoC in EV batteries is the utilization of advanced machine learning methodologies. This study utilizes two robust regression models: the ETR and the LightGBM. The selection of these models is based on their distinct characteristics, which complement each other effectively. The ETR model excels in its capacity to handle noise and mitigate overfitting, while LightGBM demonstrates remarkable speed and accuracy in making predictions. In order to guarantee optimal quality of data input into these models, we carry out an extensive preprocessing stage. This stage encompasses careful management of missing data and the removal of abnormalities, followed by advanced feature engineering techniques to extract and emphasize important information and patterns from the battery data. The preparation process plays a pivotal role in augmenting the precision and dependability of our prognostications. Subsequently, the models undergo training and fine-tuning procedures employing hyperparameter optimization approaches to attain an ideal equilibrium between precision and generalizability. A meticulous evaluation procedure is implemented, as illustrated in Fig. 2, which delineates our three-step methodology: data collection is gathering and recording information for analysis and research purposes. This stage entails collecting data from electric vehicles, encompassing crucial battery characteristics, environmental variables, and vehicle operational data. The extensive dataset serves as the fundamental basis for our subsequent study. In data preprocessing, we aim to guarantee the integrity and uniformity of the gathered data. This critical stage involves resolving missing values and eliminating noise resulting from differences in hardware or software. Subsequently, the data that has undergone processing is partitioned into distinct sets for training, testing, and validation, establishing the framework for creating and evaluating predictive models. The prepared dataset trains and fine-tunes the ETR and LightGBM models. Subsequently, the models are assessed on a testing dataset to evaluate their performance in real-world circumstances. The models' efficacy is assessed by comparing the anticipated SoC values with the observed values, employing a range of performance indicators. The proposed method is distinguished by its comprehensive approach, encompassing data gathering, preprocessing, and powerful machine learning models. This integration enables the method to deliver precise and resilient SoC estimations for EVs.

### 3.1 Machine learning algorithms

This subsection includes: an introduction to LightGBM where we provide an introduction and brief overview of LightGBM, explaining its significance and advantages as a machine learning algorithm for SoC estimation, ETR model, and proposed Flowchart for SoC estimation.

#### 3.1.1 Introduction of LightGBM

The LightGBM framework, a cutting-edge gradient-boosting methodology, leverages decision tree algorithms and integrates various novel mathematical approaches. Histogram optimization is a technique that enhances the efficiency of an algorithm by discretizing continuous feature values into bins, reducing computational complexity. In addition, LightGBM employs a distinct leaf-wise tree expansion strategy, which differs from the prevalent depth-wise methodology. Utilizing a leaf-wise technique facilitates the creation of more complex tree structures, resulting in improved accuracy and efficiency of the model when dealing with extensive datasets [34]. The methodology of Gradient Boosting Decision Tree (GBDT), a key component of LightGBM, is the iterative process of fitting decision trees to the dataset and subsequently employing gradient descent to optimize the model. The mathematical formulation of LightGBM can be delineated as follows in Equation (1):

(1) $$\varphi_{T}(x)=\sum_{1}^{T}\varphi_{t}(x)\to \varphi_{T}\in \Theta$$

Where $\varphi_{T}(x)$ represents the model's output as a function of the input *x*, expressed as a sum of the outputs from *T* individual trees ($\varphi_{t}(x)$). The convergence of this sum towards a limit function within a function space Θ is indicated, illustrating the iterative nature of model refinement in GBDT. The goal function of GBDT, which LightGBM tries to minimize, is expressed as follows in Equation (2):

(2) $$R_{t}(x)=\underset{R\in \varphi }{argmin}(F)(Y,\sum \varphi t-1(x)+Rt(x))$$

Equation (2) seeks the optimal residual $R_{t}(x)$ at each iteration (*t*), where *Y* denotes the target variable. The function *F* represents the loss function, and $\varphi_{t-1}(x)$ is the cumulative output from previous iterations. The minimization occurs overall potential base learners in the set *φ*. Within the LightGBM framework, a crucial element of the mathematical procedure entails the iterative enhancement of the model by incorporating a fresh, weak learner throughout each iteration. The process is governed by the gradient of the loss function, as theoretically represented in Equation (3):

(3) $$G_{t}=-\lambda F(Y,\varphi (t-1)(x))-{\left(\lambda \varphi_{t-1}(x)\right)}^{-1}$$

In Equation (3), $G_{t}$ denotes the gradient of the loss function at iteration *t*. The function *F* relates to the loss function, where *Y* is the target variable and $\varphi_{t-1}(x)$ is the model outcome from the last iteration. The learning rate, represented by *λ*, scales the gradient and plays an important role in the model update strategy. The term ${\lambda \varphi_{t-1}(x))}^{-1}$ provides numerical stability, particularly when $\left(\lambda \varphi_{t-1}(x)\right)$ is close to zero. This gradient is then employed in Equation (4) to minimize the loss function by finding the optimal residual function $R_{t}(x)$ for each iteration:

(4) $$R_{t}(x)=\underset{R\in \varphi }{argmin}\sum \left|G_{t}-R_{t}(x)\right|$$

The residual function, $R_{t}(x)$, is selected to minimize the absolute difference between the negative gradient $G_{t}$ and the residual, iterating over the set of candidate functions *φ*. Finally, the recursive updating of the model is encapsulated in Equation (5), which defines how the model is refined at each boosting iteration:

(5) $$R_{t}(x)=\sum R_{t-1}(x)+R_{t}(x)$$

Here, each new iteration *t* concerns adding the current residual function $R_{t}(x)$ to the sum of residuals from the last iterations $R_{t-1}(x)$, progressively refining the model. Through these equations, we show the robust mathematical framework of LightGBM, which is central to our proposed approach for SoC estimation in EV batteries. The accuracy and efficiency of LightGBM, as mathematically verified here, contribute significantly to the accuracy of our SoC predictions.

#### 3.1.2 Histogram optimization strategy

**Figure 3:**
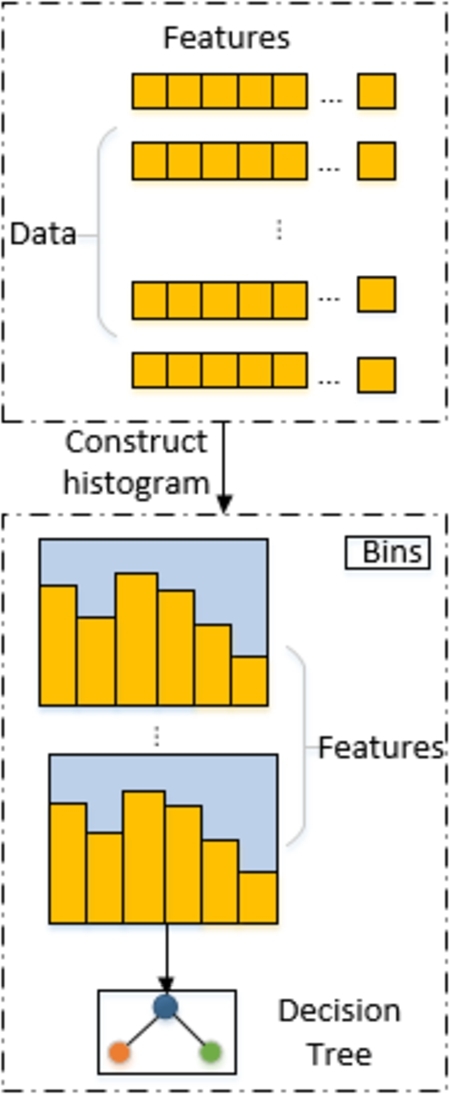
Diagram of the histogram optimization approach.

Fig. 3 illustrates the LightGBM histogram optimization technique, which discretizes continuous data into k bins during training. This simplifies calculations and speeds up the decision tree's best-split node search. The approach also helps handle noisy data by separating features with similar values into the same bin, reducing overfitting. Small bins are created from consecutive floating points, and necessary statistics are collected in the histogram during the first data scan to determine the best segmentation point [35].

#### 3.1.3 Leaf-wise growth strategy

LightGBM uses the leaf-wise growth method for decision trees, in contrast to the traditional level-wise approach. In the leaf-wise method, nodes are split based on the one with the highest information gain, leading to reduced model complexity and better overfitting prevention. While level-wise is simpler for multithreading, it may add extra computational costs and increase the risk of overfitting. On the other hand, the leaf-wise approach is more efficient in finding the best splits and performs better when there are a similar number of splits as in the level-wise technique. However, it may result in a deeper decision tree, sometimes leading to overfitting.

### 3.2 Extra tree regressor

The Extra Tree Regressor (ETR) describes a significant improvement in ensemble learning, developing from the Random Forest (RF) model originally offered by Geurts et al. [36]. The ETR technique utilizes a set of unpruned regression trees, individually generated using a conventional top-down methodology. The approach described differs from the RF model, which employs a two-step procedure involving bagging and bootstrapping for regression analysis. The ETR model employs a deterministic splitting method during the development of individual trees. In contrast to Random Forest (RF), which employs a selection process to determine the optimal split from a random subset of characteristics at each node, ETR randomly selects a split point for each feature and subsequently selects the best split among these options [37], [38]. The mathematical representation of this procedure is as follows Equation (6):

(6) $$Split_{ETR}=\underset{f,s}{\text{arg min}}\left[\text{Error}(f,s)\right]$$

In this context, the variable $Split_{ETR}$ denotes the selected split in the ETR algorithm. The symbol *f* represents a feature, while *s* represents a randomly picked split point for that particular feature. The function Error(f,s) computes the decrease in error resulting from the split. The algorithm chooses the pair of *f* and *s* that minimizes this error. During the bagging step of the RF algorithm, each tree within the ensemble contributes a vote, and the final forecast is commonly determined by taking the average of these votes. The ETR method employs a comparable strategy, however, with an ensemble of unpruned trees that are more varied. The mathematical representation of the output of the final ETR model can be succinctly expressed as Equation (7):

(7) $$Y_{ETR}=\frac{1}{N}\sum_{i=1}^{N}T_{i}(X)$$

The anticipated output, denoted as $Y_{ETR}$, is determined by the number of trees in the ensemble, denoted as *N*, the *i*-th tree in the ensemble, denoted as $T_{i}$, and the input feature vector, denoted as *X*. The ETR algorithm offers a distinctive regression strategy that balances unpredictability and accuracy by integrating mathematical components.

## 4 Battery data source

The battery data used for validation were collected from real driving trips and included various parameters such as voltage, current, temperature, discharge profile, and external environmental factors. The data was gathered from public resources about EV batteries, sourced from open sources. The dataset chosen for the study included information about the battery's performance during complete charging and discharging cycles, capturing all battery activity, including EV batteries. Data quantity, quality, and characteristics were considered when selecting the dataset. The main target of the study was the SoC estimation, and the batteries were charged using 1.5A of constant current until their capacity reached about 80%, followed by constant voltage until 100%. The discharge data were collected under various conditions, including different loading profiles (1A, 2A, and 4A), shifting ambient temperatures ($4\;{}^{\circ }$C, room temperature, and $44\;{}^{\circ }$C), and discharge voltages (2.7 V, 2.5 V, and 2.2 V), representing various climatic conditions and operating scenarios. The external temperatures, ranging from $4\;{}^{\circ }$C to $44\;{}^{\circ }$C, were also monitored, along with battery temperatures. The battery voltage was typically maintained between 3.7 V and 4.2 V to avoid deep discharge, and the total capacity fluctuated between 2Ah and 0Ah in each charging and discharging cycle. Table 2 provides the specification of the dataset used in the study, including the number of rows and columns in the dataset and the total number of features after feature engineering and selection using the proposed method.

**Table 2:** Dataset specification.

No	Description	Value
1	Total rows	1094793
2	Total Columns	48
3	Total features after feature engineering	23
4	Total features after feature selection with random forest algorithm	11

## 5 Results and discussion

This section overviews the implementation process, including data processing, feature selection, performance evaluation, and SoC estimation operating ML techniques.

### 5.1 Experimental setup

Implementing the proposed system structure and environment is the summarized proposed system experimental setup, in which the operating system, RAM, CPU, programming language, and browser are used to develop the system. Also, the operating system used Windows 10, with 16 GB of RAM. The CPU used an Intel(R) Core(TM) i5-9600 K with a speed of 3.70 GHz. The system was programmed using Python 3.8.3, and Google Chrome was used as the browser.

### 5.2 Data processing

Our study processed the battery dataset to ensure accurate SoC estimation. We integrated and cleansed the data to maintain consistency and accuracy. New features were created to capture relevant trends, and the data was normalized for effective learning. The final features selected for model training enhanced accuracy and generalization. The total dataset consists of 1,094,793 rows of battery and ambient data. The dataset is split into 90% training and 10% testing data. The training data, which comprises 942,813 rows, is used to train the ML models, while the testing data, consisting of 104,758 rows, is used to evaluate the model's performance.

**Figure 4:**
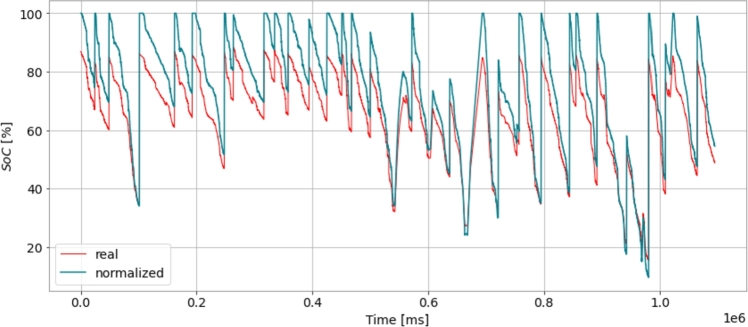
Battery SoC degradation over time.

Fig. 4 shows SoC degradation over vehicle exploitation. It compares the actual SoC values (in red) with the displayed SoC values (in blue) over time. The x-axis represents time in milliseconds, and the y-axis represents the SoC percentage. The red line represents the actual SoC values obtained from the battery, while the blue line represents the displayed SoC values, which may be the estimated or predicted SoC values. The figure provides insights into the accuracy of the SoC estimation or prediction methods used in the study by comparing the actual and displayed SoC values. Discrepancies between the red and blue lines may indicate the effectiveness of the chosen machine learning algorithms or data processing techniques in accurately estimating the battery's SoC.

### 5.3 Feature selection

**Figure 5:**
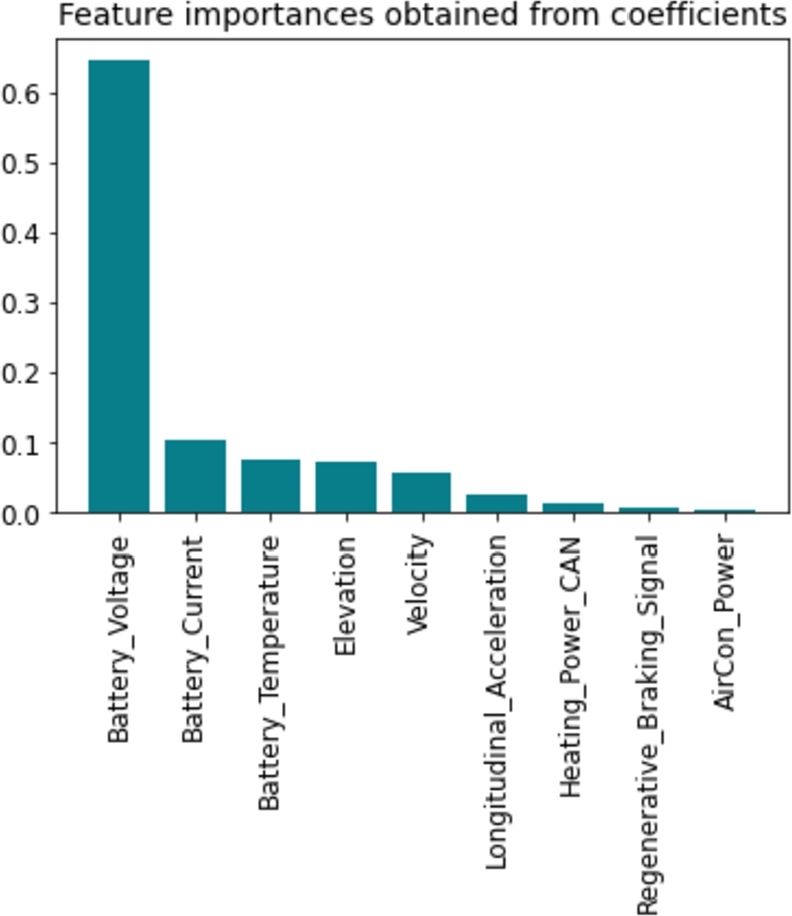
Feature importances obtained from coefficients.

Feature selection is a critical step in machine learning to identify the most relevant and informative features for model training. Our study carefully selected features that strongly impact the SoC estimation of batteries. We enhance the model's accuracy and prevent overfitting by focusing on essential characteristics and eliminating irrelevant ones. The final set of features chosen for model training has been rigorously evaluated and optimized to ensure the best possible performance in accurately estimating the SoC of the batteries. Fig. 5 visually represents the feature importances obtained from the model, indicating the priority of features based on their significance scores.

### 5.4 Evaluation metrics

In order to analyze the accuracy of the proposed prediction method more intuitively, four evaluation metrics were used to compare their performances. The evaluation metrics included the Root Mean Square Error (RMSE), Mean Absolute Error (MAE), Mean Squared Error (MSE), and $R^{2}$ [39] as shown in Equations (8), (9), (10) and (11):

(8) $${\left(RMSE\right)}_{SoC}=\sum_{i=1}^{N}\frac{1}{N}\left|(SoCi-{\overset{ˆ}{SoC}}_{i})\right|$$

(9) $${\left(MAE\right)}_{SoC}=\sum_{i=1}^{N}\frac{\left|SoCi-{\overset{ˆ}{SoC}}_{i}\right|}{N}$$

(10) $${\left(MSE\right)}_{SoC}=\frac{1}{N}\sum_{i=1}^{N}{\left(SoC_{i}-{\overset{ˆ}{SoC}}_{i}\right)}^{2}$$

(11) $${\left(R^{2}\right)}_{SoC}=1-\sum_{i=1}^{N}\frac{{\left(SoCi-\overset{ˆ}{SoCi}\right)}^{2}}{{\left(SoCi-\overset{¯}{SoCi}\right)}^{2}}$$

Where the actual SoC values are denoted as $SoC_{i}$, and the corresponding predicted SoC values are represented as ${\overset{ˆ}{SoC}}_{i}$. The summation is done over all the data points in the dataset, and the total number of data points is denoted as N. The RMSE has traditionally been the most widely used metric for regression tasks. Where the actual SoC values are denoted as $SoC_{i}$ and the corresponding predicted SoC values are represented as ${\overset{ˆ}{SoC}}_{i}$. The summation is done over all the data points in the dataset, and the total number of data points is denoted as N. The RMSE has traditionally been the most widely used metric for regression tasks. RMSE quantifies the average discrepancy between the predicted and actual SoC values by taking the square root of the average squared differences. MAE measures the average absolute discrepancy between the predicted and actual SoC values. MSE also assesses the average squared discrepancy between the predicted and actual SoC values. In the context of SoC estimation, ${\left(R^{2}\right)}_{SoC}$ represents the specific $R^{2}$ value, indicating the proportion of variance in the SoC explained by the model's predictions. A perfect fit is denoted by a value of 1, while 0 means the model fails to explain any variance. These metrics are crucial in evaluating the predictive model's accuracy and goodness of fit in SoC estimation.

Table 3 compares performance metrics for two different models: LightGBM and ETR. The performance metrics evaluated in this case are MSE, RMSE, MAE, and $R^{2}$. Both techniques successfully decrease estimation error; the suggested ETR approach achieves good efficiency with 99.83% ($R^{2}$), outperforming the methods. Table 3 indicates that the models have accurate prediction outcomes. The comparison result demonstrates that ETR performs better than LightGBM with higher $R^{2}$ values of 0.99% and 0.92%, respectively. For LightGBM and ETR, the lower MAE values are 1.39 and 0.085, respectively, and the lower RMSE values are 1.97 and 0.62, respectively. The lower MSE values for LightGBM and ETR, which perform better than LightGBM in SoC battery EV prediction, are 3.91 and 0.39, respectively. The outcome shows that the ETR model outperformed the LightGBM model in performance. Further analysis of the results showed that ETR has higher $R^{2}$ and lower MAE, RMSE, and MSE than LightGBM.

**Table 3:** Comparison of MSE, RMSE, MAE, and *R*^2^.

No	Model	MSE	RMSE	MAE	*R*^2^
1	LightGBM	3.91	1.97	1.39	0.9252
2	Extra Tree Regressor	0.39	0.62	0.085	0.9983

**Figure 6:**
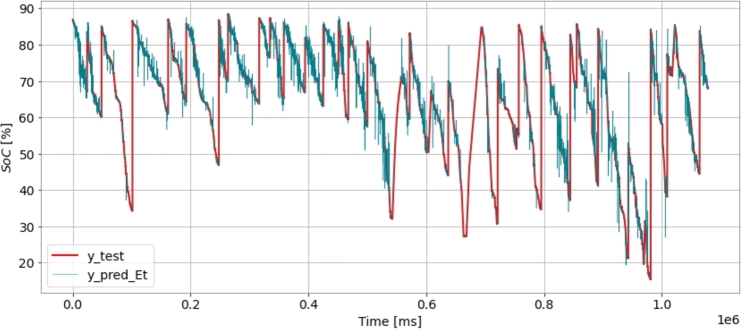
SoC prediction of the proposed model.

Fig. 6 shows an SoC battery data set before using the suggested approach. The time is shown on the x-axis, the SoC is shown on the y-axis, and the blue data points represent our predictions, which closely match the data in many cases, while the red data points show the actual values. The $y_{pred_{E}t}$ line closely follows the $y_{test}$ line; it indicates that the model's predictions are accurate and reliable.

**Figure 7:**
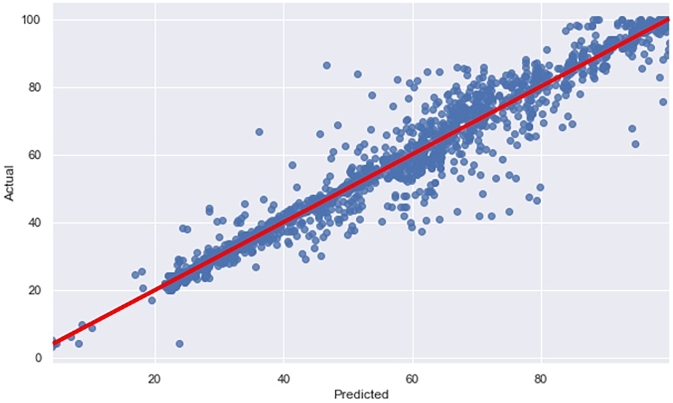
Comparison of predicted and actual SoC values using the ETR model.

In Fig. 7, the scatter plot compares the predicted SoC values and the actual SoC values obtained from the ETR model. The x-axis represents the predicted SoC values, while the y-axis represents the actual SoC values. Each point on the plot corresponds to a specific data instance, where the x-coordinate represents the predicted SoC value, and the y-coordinate represents the actual SoC value. The regression line, shown in red, provides insight into the linear relationship between the predicted and actual SoC values. This line estimates how well the ETR model's predictions align with the actual values. When the data points cluster closely around the regression line, it indicates a good fit, suggesting that the ETR model accurately predicts the SoC values. This alignment between the predicted and actual values indicates that the ETR model effectively estimates the SoC values for the given dataset.

The comparative analysis in Table 4 examines different approaches for predicting the SoC in EV batteries. These approaches include Dual Gaussian Process Regression (Dual GPR), Encoder-Decoder Networks, a diverse set of Machine Learning (ML) models, Genetic Algorithms combined with Multivariate Linear Regression, and our proposed Extra Tree Regressor (ETR) method. The assessment focuses on three primary metrics: MAE, RMSE, and the coefficient of determination $R^{2}$. The results of our investigation indicate that the ETR technique, when compared to other methods such as Dual GPR and general ML models, demonstrates superior performance. This is evidenced by the ETR method achieving the lowest MAE value of 0.085%, suggesting greater accuracy in predicting the SoC. Moreover, the root RMSE and $R^{2}$ values provide additional evidence supporting the enhanced predictive capability of our ETR (Extreme Gradient Boosting Regression) method. These results also suggest that our ETR method outperforms previous approaches in accurately predicting the SoC in EVs. The method above has the potential to yield precise real-time estimations of SoC, hence offering valuable insights for improving battery utilization and enhancing the driving range of EVs.

**Table 4:** Enhanced Comparison of Battery SoC Prediction Methods in EVs.

Ref-No	Method	Description	MAE (%)	RMSE	*R*^2^
[40]	Dual GPR	Two Gaussian processes for non-linear data modeling.	2.493	2.970	-
[41]	Encoder-Decoder Networks	Neural networks for sequential data.	0.77	-	-
[42]	ML models	Broad range of predictive algorithms.	1.9	-	-
[43]	Genetic Algorithms and Multivariate Linear Regression	Optimization with genetic algorithms and regression analysis.	-	1.0744	95
Our study	Proposed ETR Method	Unpruned decision trees for SoC prediction.	**0.085**	**0.62**	**0.9983**

## 6 Conclusion

Driving behavior in battery EVs causes considerable load changes for high-voltage batteries. The dynamic performance of the powertrain is juxtaposed with the nearly constant load imposed by the auxiliary consumers. The heating and air conditioning system incurs the most auxiliary usage, substantially reducing the vehicle's range. This study aims to assess the performance of a battery EV by analyzing data related to its battery and heating systems. This study demonstrates our machine learning approach in four steps: preprocessing, machine learning modeling, variable definition and data collecting, and a comprehensive evaluation of estimation models. The examination and comparison of machine learning models yield a significant observation: the ETR regularly exhibits superior performance compared to the LightGBM across various essential metrics, such as the coefficient of determination $R^{2}$, MAE, and RMSE. The present study examined the investigation of the ETR approach for predicting the SoC to enhance the comprehension of vehicle performance. The obtained results from this study demonstrated the efficacy of employing the ETR method for SoC prediction. The suggested methodology presents numerous benefits, such as utilizing comprehensive variables to estimate the SoC and evaluating various ML models for the most effective choice. The precise prediction of SoC facilitates improving battery capacity usage and extending the range for EVs. The study acknowledges limitations such as a small dataset and the need to consider driving habits, traffic conditions, and auxiliary loads. Future work can incorporate additional factors to enhance accuracy. Accurate trip data from EV manufacturers with diverse environmental profiles could further enrich the study's quality.

## CRediT authorship contribution statement

**Sadiqa Jafari:** Writing – review & editing, Writing – original draft, Methodology, Formal analysis, Data curation, Conceptualization. **Yung-Cheol Byun:** Supervision, Resources, Project administration, Investigation, Funding acquisition.

## Declaration of Competing Interest

The authors declare that they have no known competing financial interests or personal relationships that could have appeared to influence the work reported in this paper.

## Data Availability

Not applicable.
